# Suicide attempts in Brazil, 1998–2014: an ecological study

**DOI:** 10.1186/s12889-016-3619-3

**Published:** 2016-09-15

**Authors:** Davi Félix Martins Junior, Ridalva Martins Felzemburgh, Acácia Batista Dias, André C. Caribé, S. Bezerra-Filho, Ângela Miranda-Scippa

**Affiliations:** 1 Postgraduate Programme in Medicine and Health (PPgMS), Federal University of Bahia, Salvador, BA Brazil; 2 Department of Health, State University of Feira de Santana (UEFS), Feira de Santana, BA Brazil; 3 School of Nursing, Federal University of Bahia (UFBA), Salvador, BA Brazil; 4 Department of Humanities and Philosophy, State University of Feira de Santana (UEFS), Feira de Santana, BA Brazil; 5 Program of Mood and Anxiety Disorders (CETHA), Federal University of Bahia (UFBA), Salvador, BA Brazil; 6 VI Módulo - Departamento de Saúde, Av. Transnordestina, S/N, Bairro: Novo Horizonte, CEP: 44.036.900 Feira de Santana, BA Brazil

**Keywords:** Hospital morbidity, Suicide attempts, Deaths, Hospitalizations, Suicide

## Abstract

**Background:**

Attempted suicide is the main predictor of suicide constituting a major public health issue worldwide. It is estimated that for every completed suicide, 10 to 20 suicide attempts occur. Important part of the occurrences of suicide attempts in Brazil are registered in the hospital information system for coverage of more than 70 % allows to evaluate the extent of this problem in the country. The scope of this article is to analyse hospitalizations resulting from suicide attempts at public hospitals or services contracted out by the public health system (SUS) in Brazil from 1998 to 2014.

**Methods:**

This is an ecological study of secondary morbidity data obtained from the Hospital Information System. The overall rate of suicide attempts per 100 000 (10^5^) individuals and rates stratified by age group and sex were calculated. To measure trends, simple linear regression coefficients were calculated. The hospital mortality rate was calculated per 100 individuals.

**Results:**

The overall rate of hospitalization decreased from 1998 to 2014. The young and adult age groups had the highest hospitalization rates. Men were admitted more and the elderly had higher hospital mortality rates. The main cause of hospitalization was poisoning, accounting for 70.4 % of hospitalizations. Among the people who used poisoning by non-medical drugs as the method of attempted suicide, 58 178 (69.6 %) were men and 49 585 people who are poisoned by medical drugs (60.1 %) were women.

**Conclusions:**

Although hospitalization rates for attempted suicide have declined in Brazil, it remains a serious public health problem. Because a suicide attempt is the main predictor of suicide, studies to identify those most vulnerable to attempted suicide will help in the development of prevention strategies for mental health.

## Background

Suicide is an important public health issue. The World Health Organization (WHO) estimates that more than 1.5 million people will die from suicide in 2020 [1].

The highest reported suicide rates are in Eastern Europe [2]. In Brazil, according to the mortality information system, the rates of suicide are low (varying from 3.3/10^5^ in 1980 to 5.3/10^5^ in 2012, the last year with available data) compared with some European countries, such as Lithuania (30.72/10^5^), Russia Federation (27.40/10^5^), Belarus (25.26/10^5^), Hungary (21.53/10^5^), Ukraine (19.54/10^5^), Finland (18.45/10^5^), and Latvia (17.84/10^5^) [2].

However, little is known about suicide attempts throughout the world. It is estimated that in the countries of the WHO European Region, 150 000 people successfully commit suicide and 1 500 000 people attempt suicide annually [3]. The rate of suicide attempts that end in hospitalization is unknown in Brazil, despite the existence of a system (Hospital Information System - SIH) that records all hospital admissions due to suicide attempts in the public health system.

The SIH includes all hospitalizations within the Unified Health System (SUS), which consists of an extensive network of public and contracted hospitals throughout the country. The coverage of the system varies by state, depending on the percentage of the population that uses private health plans in each state. According to some estimates, the public health service covers approximately 70 % of total admissions in Brazil [4, 5]. Given the wide coverage of the SIH, it is a valuable source of data for planning, managing and evaluating hospital services [6] as well as for epidemiological research (the study of hospital mortality) and monitoring and auditing by the Ministry of Health [7]. Studies that use this database provide estimates of the occurrence of more serious morbidities in the population, i.e., those that require hospitalization. Hospitalization is indicated when the health risk is high, requiring specific attention and continuous monitoring, and it often involves the use of technological resources of high complexity and high cost.

Data from the SIH show that suicide attempts accounted for 153 061 admissions in people with age 10 years old or more in the period 1998 to 2014 [6]. These results reinforce the importance of attempted suicide as a leading cause of hospital admissions. Information about suicide attempts can be used to plan health services, including psychological and psychiatric evaluations, for those who make a nonlethal attempt at suicide. This is crucial for the prevention of further suicide attempts and the consequences of such attempts because a suicide attempt is by far the strongest predictor of successful suicide [8, 9]. Therefore, this study analysed the hospitalizations resulting from suicide attempts in people hospitalized in Brazil and documented by the Unified Health System in the period 1998 to 2014.

## Methods

### Study design

This research was an ecological study of a temporal series [10]. Data regarding hospitalization for suicide attempts in the SUS include individuals who came to emergency services and who were hospitalized for treatment for more than 24 h. One exception is serious cases where there was imminent threat to life but the individual was hospitalized for less than 24 h. It should be noted that in Brazil, patients who receive treatment from emergency services but are under observation for less than 24 h are considered outpatients and are not entered into the hospital system’s statistics. If hospitalization of more than 24 h is necessary, the patient is given a tab for hospitalization (AIH), which allows the patient to be admitted to a public health unit or a hospital contracted by the SUS. The AIH is used to identify patients and the services that they received under the scheme of hospitalization and ensures access to a clinic, as well as payment to service providers during hospitalization.

Data on all hospital admissions between 1998 and 2014 were collected and coded according to the International Statistical Classification of Diseases and Related Health Problems ICD-10 as codes X60.0 to X84.0, which correspond to suicide or self-inflicted injuries. The database used to collect data at admission was the MS/SVS/DASIS-SIH/DATASUS 2014 database [6]. The data were stratified by gender (male and female) and seven age groups: 10–14 years (children), 15–24 years (young), 25–34 years (young adults), 35–44 years and 45–54 years (adults), 55–64 (middle-age) and 65 years or older (elderly). The first purpose of this study was to describe trends in the rate of hospitalization due to suicide attempts between 1998 and 2014. The overall rate of suicide attempts per 100 000 (10^5^) individuals and the rates stratified by age and sex were calculated.

### Statistical analysis

To measure trends, simple linear regression coefficients were calculated. To examine the nature and statistical significance, the simple linear regression method (y = a + *b*x) was employed, with time (in years) as the independent variable and suicide rate (overall and stratified by sex and age group) as the dependent variable; the “a” and “b” values were obtained by the least squares method. The estimated slope parameter b, 95 % confidence interval and associated p-value were calculated. The slope parameter b is an estimate of the per unit change in suicide attempt rate, and the p-value indicates the significance of the relationship between the rate and the year.

The hospital mortality rate was also calculated (number of hospitalizations for intentional self-harm with the intention of death/by total admissions for self-harm * 100). We also examined the methods used to produce injuries that led to hospital admission. Injuries were classified into the following major categories: poisoning by medical drugs (X60-X64), poisoning by non-medical drugs (X65-X69), firearms (X72-X74), fire (X76), cutting (X78-X79), jumping (X80), other unspecified means (X84), and a residual group of other types of injury (X70, X71, X75, X77, X81, X82 and X83). In the last group, each category contributed to less than 1 % of hospitalizations, except category X83, which accounted for 1.1 % of hospitalizations.

## Results

Between 1998 and 2014, 153 061 hospitalizations for suicide attempts in people with 10 years or more of age were recorded in public hospitals and hospitals contracted by the SUS, of which, 93 634 (61.2 %) hospitalizations were in male subjects, and 59 426 (38.8 %) hospitalizations were in female subjects, and sex was not reported in one case. A total of 6 906 (4.5 %) cases were children, of whom 53.1 % (3 667) were female, A total of 37 596 hospitalized patients 9 249 (24.6 %) were young people (15–24 years old), The volume of admissions increased to 37 596 in young adults (25–34 years old) decreasing successively in subsequent age groups until 8 068 admissions, in the elderly, people 65 and older. In all age groups predominated men hospitalized for attempt suicide ranging from 54.1 % in young people (15–24 years old) to 67.7 % in middle age group (55–64 years old), except in children.

**Fig. 1:**
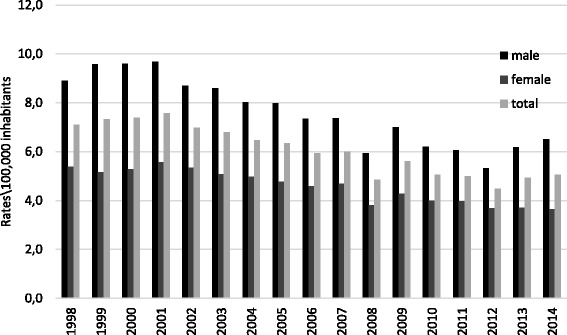
Rates of hospitalization for attempted suicide according to sex, Brazil, 1998–2014

The rate of hospitalization for attempted suicide increased from 7.1 cases/10^5^ inhabitants in 1998 to 7.6/10^5^ in 2001, oscillating in the following years and then reaching 5.1 cases/10^5^ in 2014, the last year of the study series, (Fig. 1). The hospitalization rate for suicide attempts decreased during the study period β (95 % CI) = −0.019 (−0.023--0.015) *p* <0.001, *r*^2^ = 0,885).

The rates of hospitalization for attempted suicide decreased over the study period for both sexes: men β (95 % CI) = −0.026, (−0.032--0.020), *p* <0.001 *r*^2^ = 0,849), peak 9.7/10^5^ in 2001, and women β (95 % CI) = −0.013, (−0.015--0.011), *p* <0.001 *r*^2^ = 0,903), peak 5.6/10^5^ in 2001. After stratification by gender, the hospitalization rate was consistently higher in males, with an average ratio of men to women of 1.6. Among both males and females, declining hospitalization rates over the study period were observed in all age groups. The highest average male/female ratio was observed in the 55–64 year-old age group (2.4) and the lowest in young, (1,2).

**Fig. 2:**
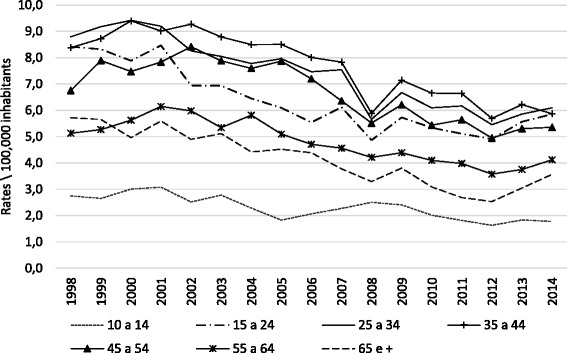
Rates of hospitalization for attempted suicide according to age group, Brazil, 1998–2014

The rate of hospitalization also decreased in all age groups during the study period. Children exhibited a lower rate of hospitalization, with fewer than 2.0 cases/10^5^ inhabitants and a gradual reduction over time β (95 % CI) = −0.007, (−0.010--0.004), *p* <0.001 *r*^2^ = 0,660). Young β (95 % CI) = −0.021, (−0.028--0.015), *p* <0.001 *r*^2^ = 0,755); young adults β (95 % CI) = −0.025, (−0.030--0.020), *p* <0.001 *r*^2^ = 0,879); adults from 35 to 44 years old β (95 % CI) = −0.023, (−0.029--0.017), *p* <0.001 *r*^2^ = 0,801); adults from 45 to 55 years old β (95 % CI) = −0.019, (−0.026--0.012), *p* <0.001 *r*^2^ = 0,701); on middle-age group (55 to 64 years old) β (95 % CI) = −0.013, (−0.018--0.009), *p* <0.001 *r*^2^ = 0,736); The elderly had the second lowest hospitalization rate, averaging 4.2 cases/10^5^ inhabitants β (95 % CI) = −0.019, (−0.024--0.015), *p* <0.001 *r*^2^ = 0,862), (Fig. 2).

**Fig. 3:**
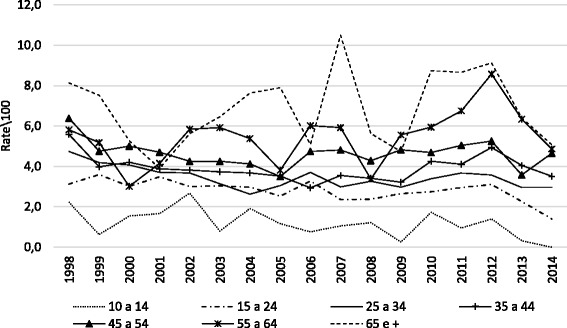
Hospital mortality rates due to suicide attempt according to age group, Brazil, 1998–2014

The hospital mortality rate was found to increase with age. The average rate in children was 1.2 deaths/100 inhabitants increasing and reaching 6.8 deaths/100 inhabitants in the elderly. The hospital mortality rates in the elderly group rates varied greatly throughout the study period, starting with 8.1 deaths/100 inhabitants in 1998, peaking at 10.5 deaths/100 inhabitants in 2007 reducing to 5.1 deaths/100 inhabitants in the last year of the study period (Fig. 3).

From suicide attempts that end in hospitalizations, poisoning accounted for 70.4 %, and in 8.1 % of cases, the methods used were not specified. Among the people who used poisoning by non-medical drugs as the method of attempted suicide, 58 178 (69.6 %) were men and 49 585 people who are poisoned by medical drugs (60.1 %) were women. Among those who used firearms as the method of suicide attempt, 88.6 % were men. The method of shoot oneself was predominately used by women (59.4 %) (Table 1).

**Table 1:** Methods used in suicide attempts^a^, Brazil, 1998–2014

Means	n	% male	% female	% total
Poisoning by non-medical drugs (X65 - X69)	58 178	69,6	30, 4	38, 0
Poisoning by medical drugs (X60 - X64)	49 585	39, 9	60, 1	32, 4
Cutting/piercing with objects (X78 + X79)	12 719	81, 8	18, 2	8, 3
Firearms (X72 – X74)	7 716	88, 6	11, 4	5, 0
Other means ^a^	4 569	68, 9	31, 1	3, 0
Fire (X76)	4 008	40, 6	59, 4	2, 6
Jumping (X80)	3 882	76, 0	24, 0	2, 5
Other unspecified means (X84)	12 403	67, 6	32, 4	8, 1
Total	153 060	61, 2	38, 8	100, 0

Source: SIH/DATASUS

^a^1 case sex ign

## Discussion

Our data show a general decline in hospitalization rates for suicide attempts in both sexes in Brazil (7.1 per 100 000 inhabitants in 1998 to 5.1 per 100 000 in 2014) and in all age groups.

We believe that the decrease in the hospitalization rate for suicide attempts during this period was due to the expansion of public health services available to the population, particularly primary health care services and the implementation of specialized care centres for mental health.

Approximately 50 % of attempts were carried out by young and young adults, of 15 to 34 years of age. The rate of hospital mortality increased with age, poisoning was the method most often used in suicide attempts requiring hospitalization in both sexes, accounting for 70.4 % of suicide attempts. Distributed according to gender, there was a predominance of women among those who used medical drugs for poisoning (60.1 %), while for those who used non-medical drugs, there was a predominance of men (69.6 %).

Men more commonly used lethal suicide attempt methods, such as weapons / blunt objects and firearms (81.8 and 88.6 %, respectively) compared with women. However, shoot oneself predominated among women (59.4 %). This study examined data on hospitalizations at public health units only, and included hospitalizations of more than 24 h in duration for treatment or observation.

Men were found to have higher rates of hospitalization for attempted suicide compared with women for each year of the period investigated. This pattern is similar to that for suicide mortality, as more men than women die [11–13]. This is a predominant phenomenon in men in most countries, except some Asian countries such as China and is the result of differences in the methods of suicide attempt used between males and females [14]. In fact, in one study, the methods of attempted suicide were hanging or gunshot for seven out of ten boys and drug overdose for nine out of ten girls [15]. By contrast, more women attempt suicide than men [16, 17]. However, women tend to use less effective and gentler methods in their suicide attempts than men, which less frequently require hospitalization. Men attempt suicide using more lethal methods, which reduces the probability of survival after the attempt. It is possible that men are more exposed to social pressure and that masculinity involves behaviours that predispose men to suicide, including greater access to lethal instruments and firearms [18].

A downward trend in hospitalization rates was observed in all age groups studied regardless of sex, although there were different risk factors for each age group. Young people and adults had the highest rates of hospitalization due to attempted suicide in the study period, accounting for over 80 % of hospital admissions. In fact, self-injury and suicide are known to be major public health problems among adolescents, and self-injury rates are high in adolescents, and suicide is the second most common cause of death in young people around the world [19]. Moreover, among the elderly, those who have no gainful employment or other source of income and those who have lost one or more family members or friends have an increased risk of suicide [20–22].

The causes of suicidal behaviour are still not completely understood, but genetic, social and environmental factors are involved (diathesis-stress model). Previous suicide attempts and having relatives or acquaintances who attempted or committed suicide are also associated with suicidal behaviour [23]. Affective disorders are common in those who attempt suicide as are the comorbidities of substance abuse and personality disorders [24]. In addition, some environmental stressors, such as family breakdown, emotional neglect and sexual abuse during childhood, are also cited in the literature as risk factors for suicidal ideation and lifelong suicide attempts. [25–31] Some form of mental disorder is present in 71 to 97 % of people who commit suicide, with mood disorders being the most frequent, followed by disorders related to substance abuse (alcohol in particular) schizophrenia and personality disorders [32].

The methods chosen for suicide attempts should also be considered because they are an important variable that help to explain the differences in hospitalization rates. A study conducted in Poland that assessed suicide attempts by various methods showed that the most common method was intoxication with medical drugs (42.31 %) [16]. In a review of methods used to commit suicide in Asia, hanging and poisoning with solid/liquid substances (mainly pesticides) were the predominant methods [33]. In Canada, the profiles of the methods used to commit suicide among young people and adolescents changed from 1980 to 2008. There was a decrease in the use of firearms and an increase in death by asphyxiation, which has become the most common method of suicide in children and adolescents in Canada [34].

Moreover, in a recent study that investigated self-injury among children in the United States in a sample taken from emergency departments across the country, poisoning was revealed to be the most commonly used method of attempted suicide that end in hospitalization, accounting for approximately 70 % of cases [35]. In Italy, poisoning was found to be the most commonly used method of attempted suicide, accounting for 69.3 % of cases [36].

These previous findings are consistent with those of the current study, as 70.4 % of suicide attempts that end in hospitalization were due to poisoning in this study. Violent methods, such as the use of firearms, accounted for 5 % and hanging less than 1 %. Therefore, less-lethal methods were predominantly used.

The database of hospital admissions has data on hospital mortality, which included relevant information that was incorporated in the manuscript. We found that the mortality rate was higher in the elderly group than in the other age groups. Some of the possible reasons for the observed pattern is that elderly can make use of more lethal methods and, perhaps, because the clinical injuries secondary to suicide attempt in this age group are more deleterious [37], thus, generating a closer relationship between suicide attempts and successes.

Young people aged 15 to 34 years old accounted for 49.2 % of suicide attempts, which was similar to reports of other studies conducted in Brazil [38, 39], and indicates a higher risk of suicide attempt in this age group.

It is important to highlight that suicidal behaviour is very deleterious. Although, in some cases, the victim can escape death with little or no sequelae and can return to their usual activities, in other cases, serious sequelae can develop that prevent the individual from leading a normal life and that generate long-term costs to the family and the State for the maintenance and care of that individual. These consequences contribute to a reduced quality of life for those individuals through the generation of additional social and affective problems [40].

### What this study adds

This study was a temporal trend analysis of hospital admissions due to attempted suicide in Brazil recorded in the Unified Health System from 1998 to 2014. We used linear regression analysis to assess trends in attempted suicide during the study period. We described temporal trends in attempted suicide rates according to sex and age, and we described the proportional distribution of the suicide attempt methods used by sex. This study reviewed all cases of suicide attempts resulting in hospitalization that were recorded in the hospital information system since 1998, the first year with available data.

### Limitations

Possible limitations of our data should be discussed. We only included patients who were seen at public hospitals or hospitals contracted by the SUS; therefore, we did not obtain data on patients who were hospitalized at private hospitals, which are either paid directly or through private health insurance plans. It is likely that numerous patients who deliberately self-harmed and presented to public emergency units were not included in this study, as some patients are not formally admitted, especially those who do not occupy a bed, and who are treated rapidly in an emergency unit and discharged on the same day [41]. Furthermore, it is known that some individuals who attempt suicide remain in their familial or social environment afterwards and do not receive any specific health care; therefore, data about these suicide attempts were not included in the official statistics [42].

There is much discussion in the literature on the underreporting of suicide, which is probably the largest source of error in determining the rates of suicide and suicide attempts. However, if underreporting is more or less random for variables such as men and women, young adults and older adults, etc., then the relative differences in the trends in suicide attempts for any variable may not be seriously biased. Underreporting can cause a serious problem if it varies systematically based on the time period, age or gender. Therefore, it is necessary that studies be conducted to confirm that underreporting occurs randomly. Thus, the data used in this study reflect only a fraction of the real number of hospitalizations due to suicide attempt in Brazil. We can infer that the real rate of hospitalization due to suicide attempt and the trend in suicide attempts in the general population in Brazil has been underestimated.

## Conclusions

Hospitalization rates for suicide attempts were found to have decreased during the study period.

It is known that increased access to mental health services is protective against suicide and suicide attempts. Access to more lethal methods could inversely increase the number of deaths by influencing the rate of suicide attempts.

Highest rates were observed in males. The young and adult age groups were the most frequently hospitalized for suicide attempts, and the elderly age group had the highest mortality rate. Poisoning accounted for 70.4 % of suicide attempts that end hospitalization, and among men, poisoning was predominantly performed using non-medical drugs (69.6 %), while among women, poisoning was predominantly performed using medical drugs (60.1 %).

As it is known that a previous suicide attempt is the strongest predictor of suicide, there is a great need to advise victims to seek treatment, i.e., appropriate monitoring with psychiatric and psychosocial therapies, to avoid the risk of repetition of the act, given that prevention of this type of disorder through diagnosis and treatment is a priority in psychiatric practice. Failing to refer a victim to a specialized service can result in subsequent suicide attempts, thus, increasing suicide rates.

Preserving life should be the ultimate purpose of the SUS, and data on suicide attempts indicate that there is much work to be done to improve survival rates and to avoid future attempts at suicide in all age groups.

## Abbreviations

AIH: Authorization Form for Hospital Admission
DATASUS: Information Technology Department of the SUS
IBGE: Brazilian Institute of Geography and Statistics
ICD-10: International Statistical Classification of Diseases and Related Health Problems
MS / SVS / DASIS-SIH / DATASUS: Ministry of Health / Secretariat of Health Surveillance / Department Analysis of Health Situation / Hospital Information System
SIH: Hospital Information System
SUS: Unified Health System
WHO: World Health Organization
